# Aryl hydrocarbon receptor signaling in male fertility: Protective role of resveratrol and disruptive effects of CH223191 in adult male rats

**DOI:** 10.14202/vetworld.2025.1274-1287

**Published:** 2025-05-21

**Authors:** Ghadeer Sabah Bustani, Hasan Falah Kashef Alghetaa

**Affiliations:** 1 Department of Physiology, Biochemistry and Pharmacology, College of Veterinary Medicine, University of Baghdad, Baghdad, Iraq; 2 Department of Anesthesia, Faculty of Medical Technologies, The Islamic University, Najaf, Iraq

**Keywords:** antioxidant capacity, aryl hydrocarbon receptor, resveratrol, CH223191, oxidative stress, sperm DNA fragmentation, spermatogenesis

## Abstract

**Background and Aim:**

The aryl hydrocarbon receptor (AhR) plays a pivotal role in spermatogenesis through its regulatory functions in redox balance and gene expression. This study aimed to investigate the effects of resveratrol (RES), a polyphenolic AhR modulator, and CH223191, a selective AhR antagonist, on male reproductive function in rats by assessing sperm quality, oxidative stress, testicular histopathology, and *AhR* gene expression.

**Materials and Methods:**

Forty adult male rats were randomly divided into four groups: (i) Control, (ii) dimethyl sulfoxide (vehicle), (iii) RES (100 mg/kg i.p., twice weekly), and (iv) AhR¯ (CH223191, 10 mg/kg i.p., twice weekly), treated for 60 days. Post-treatment, sperm motility, survival, viability, and DNA fragmentation were evaluated. Total antioxidant capacity (TAC), malondialdehyde (MDA) levels, testicular histopathology, and *AhR* gene expression quantitative polymerase chain reaction (qPCR) were analyzed.

**Results:**

RES significantly enhanced sperm motility, survival, and viability, reduced DNA fragmentation, and increased TAC while decreasing MDA levels. Histologically, RES preserved normal testicular architecture. In contrast, AhR inhibition through CH223191 led to marked reductions in sperm quality, elevated oxidative stress, increased DNA fragmentation, and severe testicular degeneration. qPCR analysis revealed upregulation of AhR expression in the RES group (fold change: +23.1%) and significant downregulation in the AhR¯ group (fold change: −72.6%), indicating differential modulation of AhR signaling pathways.

**Conclusion:**

RES positively modulates AhR activity, safeguarding testicular structure and enhancing sperm quality through antioxidant and anti-apoptotic mechanisms. Conversely, AhR antagonism disrupts spermatogenesis, underscoring the receptor’s essential role in male fertility. These findings suggest the therapeutic potential of AhR-targeting agents like RES in ameliorating male reproductive dysfunctions associated with oxidative stress and xenobiotic exposure.

## INTRODUCTION

The aryl hydrocarbon receptor (AhR) is a multifunctional cytosolic receptor widely expressed in numerous tissues throughout various stages of development [1]. This extensive expression highlights AhR’s essential role in maintaining physiological homeostasis and mediating cellular responses to environmental stimuli [2]. Its presence across diverse tissues and life stages further emphasizes its importance in fundamental biological processes [3]. Functionally, AhR is a ligand-activated transcription factor involved in regulating a broad spectrum of physiological and developmental pathways, including cell proliferation, xenobiotic metabolism, and developmental signaling cascades [4].

Seminal research in the 1970s identified AhR as a key player in xenobiotic metabolism, demonstrating that its activation induces cytochrome P450 enzymes, thereby enhancing the biotransformation and solubility of foreign chemicals, facilitating their elimination from the body [5]. While a previous study by Esakky *et al*. [6] focused primarily on its role in metabolism and toxicity, more recent investigations have revealed its involvement in the regulation of reproductive and immune functions. Specifically, AhR is now recognized as a critical regulator of reproduction, with implications for fertility, embryonic development, and hormone signaling [7, 8]. Its interaction with both endogenous and exogenous ligands influences gene expression and contributes significantly to maintaining reproductive homeostasis [9–11].

Experimental findings have demonstrated that AhR activation modulates genes involved in spermatogenesis. In rats, AhR expression within the seminiferous tubules is localized to primary pachytene spermatocytes during stages VII-XI and to round spermatids during stages II-XIV of the spermatogenic cycle. In contrast, AhR and its dimerization partner ARNT are expressed throughout all seminiferous tubule stages in the human testis [12–14]. In addition, AhR-deficient mice exhibit a dose-dependent decline in male fertility, sperm count, and the weights of seminal vesicles and dorsolateral prostate, underscoring the receptor’s pivotal role in male reproductive function [15].

Resveratrol (RES) has been identified as a partial antagonist of AhR, with one mechanism involving suppression of *AhR* gene expression. It also inhibits the activity of CYP1A1 and CYP1B1, leading to reduced reactive oxygen species (ROS) production [16]. This antioxidative effect is particularly significant, as lipid peroxidation of polyunsaturated fatty acids compromises membrane fluidity and the functionality of membrane-bound enzymes and ion channels, ultimately affecting sperm motility [17]. RES protects cells against DNA damage and apoptosis by modulating the expression of both anti- and pro-apoptotic factors, thereby enhancing cellular antioxidant defenses. Studies by Banerjee *et al*. [18] and Mongioì *et al*. [19] have shown that RES inhibits cytochrome P450 enzymatic activity and transcription through AhR antagonism, suggesting its potential in reducing cellular exposure to carcinogens.

Moreover, RES facilitates the translocation of AhR to the nucleus and promotes its binding to DNA response elements, thereby regulating gene expression [20, 21]. Interestingly, despite this nuclear translocation, RES inhibits downstream transactivation processes, resulting in multiple forms of cell death, including necrosis, apoptosis, and pyroptosis [22, 23]. This inhibitory capacity extends to key molecular targets such as CYP1A1 [24] and interleukin-1β [25, 26]. Additional studies have further demonstrated that RES acts as a competitive antagonist for AhR, effectively competing with other ligands for receptor binding and mitigating their associated toxic and carcinogenic effects. These findings support the notion that RES modulates AhR activity and can attenuate the deleterious effects of harmful ligands through receptor antagonism or selective activation [26].

While the AhR is traditionally recognized for its role in xenobiotic metabolism, emerging evidence has highlighted its functional relevance in reproductive physiology, particularly in spermatogenesis and testicular homeostasis. AhR expression within specific stages of the seminiferous epithelium suggests its involvement in the regulation of germ cell development. However, despite growing interest, the mechanistic interplay between AhR signaling and male fertility remains poorly understood, particularly regarding its redox-mediated modulation and influence on chromatin integrity. Although prior studies have examined AhR knockout models, comprehensive evaluations comparing AhR activation versus pharmacological inhibition on sperm quality, oxidative stress, and histopathological outcomes are lacking. Furthermore, the potential protective role of RES – a known AhR modulator with antioxidant properties – against chemically induced reproductive disruption has not been systematically characterized *in viv*o. These gaps hinder a full understanding of AhR’s functional significance in male reproduction and limit the development of targeted interventions to mitigate infertility associated with oxidative damage or environmental toxicants.

The primary aim of this study was to investigate the modulatory effects of AhR on male reproductive functions by evaluating the outcomes of its activation and inhibition using RES or CH223191, respectively, in adult male rats. This study specifically assessed sperm motility, viability, DNA fragmentation, antioxidant status, total antioxidant capacity (TAC), lipid peroxidation, malondialdehyde (MDA), testicular histopathology, and *Ah*R gene expression. By delineating the opposing biological effects induced by AhR activation and antagonism, the study sought to elucidate the receptor’s role in maintaining testicular integrity and spermatogenic efficiency, thereby contributing to the development of potential therapeutic strategies for oxidative stress-induced male infertility.

## MATERIALS AND METHODS

### Ethical approval

The study protocol was approved by the Institutional Animal Care and Use Committee of the University of Baghdad (Approval No. N.P.G. 685), ensuring adherence to ethical standards. Ethical considerations were paramount throughout all stages of the study, and efforts were made to minimize potential harm to the experimental animals.

### Study period and location

This study was conducted from March 2023 to October 2023 at the College of Veterinary Medicine, University of Baghdad.

### Experimental animals

Forty adult male rats weighing between 225 g and 275 g were used in this experiment. The experimental procedures were initiated on March 23, 2023, following a 2-week acclimatization period. Rats were randomly assigned to cages, with six animals housed in each cage at the Animal House of the College of Veterinary Medicine, University of Baghdad. All animals had unrestricted access to food and water throughout the experimental period.

### Experimental design

Forty male rats were randomly assigned to four groups: (1) Control Group (n = 10), receiving no treatment; (2) dimethyl sulfoxide (DMSO) Group (n = 10), receiving dimethyl sulfoxide intraperitoneally; (3) RES Group (n = 10), treated with 100 mg/kg RES intraperitoneally twice weekly for 60 days [27]; and (4) AhR¯ Group (n = 10), treated with CH223191, an AhR antagonist, at 10 mg/kg intraperitoneally twice weekly for 60 days [28]. At the conclusion of the 60-day experimental period, all animals were sacrificed, and the tail of the epididymis, blood, and testes were collected for further assessment.

### Treatment preparation

A RES solution was prepared at a concentration of 100 mg/kg body weight, containing 400 mg of RES (sufficient for 20 animals) in a mixture of 2 mL DMSO and 2 mL water. The resulting solution was accurately mixed for homogeneity using a vortex mixer, and each rat received a dose based on its weight (1 μL/1 g B.W.) [27]. Similarly, a CH223191 solution was prepared at a concentration of 10 mg/kg body weight by dissolving 40 mg of CH223191 (sufficient for 20 animals) in a 2 mL DMSO mixture. Each solution was vortexed thoroughly to ensure homogeneity before intraperitoneal administration, and each rat received a dose based on its weight (0.5 μL/1 g B.W.) of the resulting solution [28].

### Animal preparation

On day 60, rats were anesthetized using ketamine (90 mg/kg B.W.) and xylazine (40 mg/kg B.W.) before tissue collection. Post-anesthesia, the left tail of the epididymis, testis, epididymis, and blood were collected, and the animals were subsequently euthanized. The left cauda epididymis was rinsed and incubated in 2 mL of saline at 37°C to release spermatozoa. The sperm were cut using anatomical micro-scissors to extract the sperm for further evaluation, following the methodology outlined by Ngaha Njila *et al*. [29].

### Sperm motility

For the assessment of sperm motility percentage, a 10 μL aliquot of the sperm suspension was placed on a dry and pre-warmed slide. Subsequently, the sample was evaluated under a light microscope (Lab, USA) at 400× magnification [30].

### Assessment of sperm survival

Sperm survival was assessed at different time intervals (2, 4, 6, 8, 10, and 15 min) following the collection of sperm samples. A 10 μL aliquot of the sperm suspension was placed on a clean and warmed slide. The slide was then covered with a coverslip at a controlled warmed temperature. At each specified time point, a 10 μL aliquot of the sample was analyzed under a light microscope (Lab, USA) at 400× magnification.

### Sperm viability

**Figure 1:**
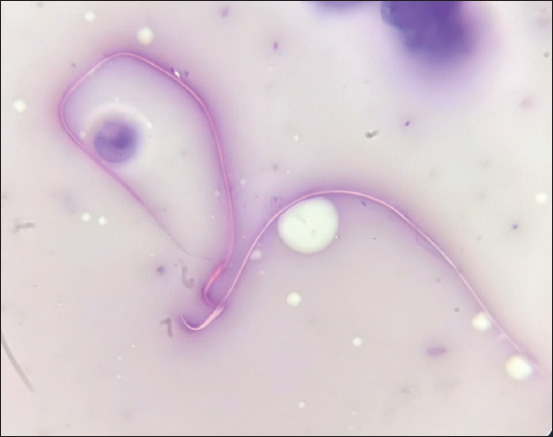
Assessment of sperm viability using eosin-nigrosine staining- A comparative analysis of live and dead sperm.

Sperm viability was assessed using eosin-nigrosine (EN) dye, as previously reported by Felipe-Pérez *et al*. [31] and Murcia-Robayo *et al*. [32]. In brief, a 10 μL aliquot of raw sperm was mixed with 20 μL of EN and gently mixed for 10 s. The resulting mixture was evenly spread on a dry, pre-warmed slide before being allowed to desiccate on a slide warmer set at 45°C [17]. Subsequently, the slides were examined under a microscope at 400× magnification, with at least 200 spermatozoa examined. Viability was determined by differentiating pink-stained (non-viable) from unstained (viable) spermatozoa under light microscopy. This methodology provided a quantitative measure of sperm viability, offering valuable insights into the overall health and functional status of the sperm population (Figure 1).

### Fixation process

For fixation, a 10 μL sperm aliquot was smeared onto a clean slide, air-dried briefly, and fixed in a 3:1 methanol-glacial acetic acid solution for 5 min. The slide was left to air-dry completely after fixation. This meticulous procedure ensures the preservation of sperm morphology on the slide, setting the stage for accurate microscopic examinations and subsequent analyses [33].

### Sperm DNA fragmentation test

**Figure 2:**
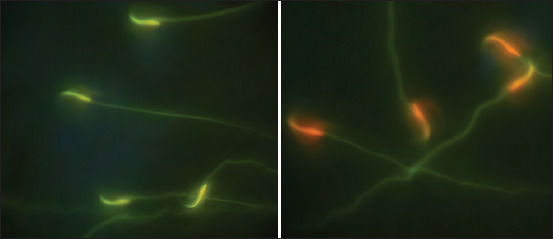
Assessment of sperm chromatin integrity and condensation using acridine orange staining - A comparative analysis of condensed chromatin (green fluorescence) and denatured chromatin (red fluorescence).

Following the fixation process, the specimens were immersed in a trough containing a working solution of acridine orange stain (0.01). After a 2-min staining, the slides were gently washed with distilled water and subsequently air-dried. The prepared slides were then subjected to a thorough examination using a fluorescent microscope [33]. Under normal conditions, sperm nuclei manifest distinct colors when observed under a fluorescence microscope, with green fluorescence indicating binding to native DNA and signifying well-organized and condensed chromatin. Conversely, red fluorescence is observed when the dye binds to denatured, fragmented DNA, indicating less condensed and fragmented chromatin. This differential coloration serves as a qualitative marker, allowing for the assessment of sperm chromatin integrity and condensation status, which are crucial factors in determining sperm quality and fertility potential (Figure 2) [34].

### TAC

The TAC Assay Kit (Solarbio Life Sciences, China; Cat. No: BC1315) was used to assess total antioxidant levels in biological samples. The protocol involved the use of a spectrophotometer reader and various components, including extraction solution, Reagent I, Reagent II, Reagent III, and a FeSO_4_ standard solution. Samples, comprising serum and tissue, underwent specific preparation steps before analysis. The assay procedure consisted of diluting the FeSO_4_ standard solution, preheating the spectrophotometer, and adding the reagents as specified. The resulting absorbance values were utilized to create a standard curve and calculate the TAC.

### MDA

The MDA Content Assay Kit (Solarbio Life Sciences, China; Cat. No: BC0025) was used to assess lipid peroxidation. The kit includes extraction reagent, liquid Reagent I, powder Reagent II, and MDA. The MDA working reagent was prepared by dissolving Reagent I in Reagent II, followed by heating at 70°C or ultrasonic treatment. The experimental procedure involved sample preparation from cell serum and tissues, followed by centrifugation to obtain the supernatant. The determination was performed by incubating the mixture of MDA, the sample, and Reagent III at 100°C for 60 min. After cooling, the absorption at 532 and 600 nm wavelengths was measured using a spectrophotometer. MDA content was calculated using specific formulas based on protein concentration.

### Histopathological examination of testicular tissue

Histopathological examination of the testicular tissue involved the excision of the testis, which was subsequently opened longitudinally and preserved in a 10% formalin solution until histological sections were prepared. Following established protocols [35], tissue sections were prepared meticulously. Immediately after the removal of tissue samples from the organs, specimens were fixed in 10% buffered formalin for 48 h at room temperature (22°C–25°C). Subsequent procedures included graded dehydration in alcohol, clearing in two stages of xylene, and embedding in liquid paraffin at 56°C for 2 h. The tissues were sectioned at a thickness of 5 μm using a microtome. The final step involved dewaxing and staining with Eosin and Harris Hematoxylin. Tissue sections were examined using the 4×, 10×, and 40× objectives of light microscopy, providing a detailed assessment of the histological features.

### Statistical analysis

The statistical analysis of the collected data was performed using GraphPad Prism 9, using the one-way analysis of variance method. This robust statistical approach was instrumental in examining the significant differences among the groups, particularly in the context of various experimental conditions and treatments. The analysis also included the Holm-Sidak correction for multiple test comparisons, considering a significance threshold of p < 0.05. Significance levels were denoted as follows: *for p < 0.05, **for p < 0.01, and ***for p < 0.001.

## RESULTS

The study evaluated a range of parameters, including sperm characteristics, oxidative stress markers, histopathological features, and *AhR* gene expression.

### Sperm motility

**Figure 3:**
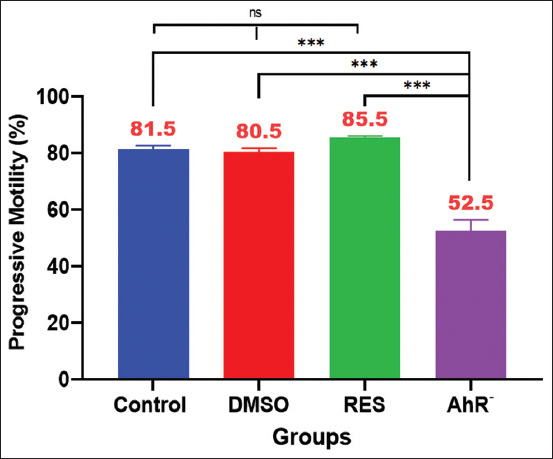
Sperm progressive test %. Values are expressed as the means, and error bars represent standard error. DMSO: Administered dimethyl sulfoxide intraperitoneally twice weekly for 60 days. RES: Receiving 100 mg/kg resveratrol intraperitoneally twice weekly for 60 days. Ahr¯: Provided with CH223191, intraperitoneally at 10 mg/kg twice weekly for 60 days. *Denotes differences between groups motility, p < 0.05. Ahr=Aryl hydrocarbon receptor, RES=Resveratrol, DMSO=Dimethyl sulfoxide. *p < 0.05, ** p < 0.01, and ***p < 0.001.

The results of progressive sperm motility are presented in Figure 3. A marginal decrease in motility was observed in the DMSO group, which was not significantly different from the control group. In contrast, the AhR¯ group, receiving CH223191, exhibited a significant reduction in sperm motility compared with all groups. Notably, rats treated with 100 mg/kg RES demonstrated a significant increase in sperm motility compared with the AhR¯ group (Figure 3).

### Assessment of sperm survival

**Figure 4:**
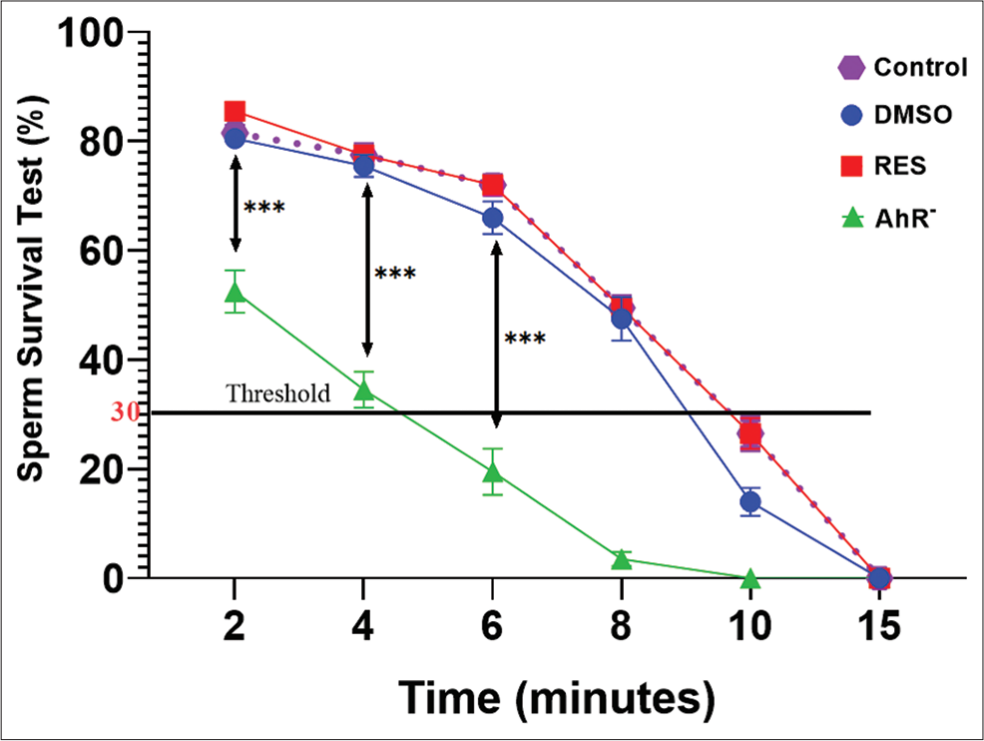
Sperm survival test %. Values are expressed as the means, and error bars represent standard error. DMSO: Administered dimethyl sulfoxide intraperitoneally twice weekly for 60 days. RES: Receiving 100mg/kg resveratrol intraperitoneally twice weekly for 60 days. Ahr¯: Provided with CH223191, intraperitoneally at 10 mg/kg twice weekly for 60 days. *Denotes differences between groups motility, p < 0.05. Ahr=Aryl hydrocarbon receptor, RES=Resveratrol, DMSO=Dimethyl sulfoxide. *p < 0.05, ** p < 0.01, and ***p < 0.001.

Regarding sperm survival (Figure 4), the analysis revealed significant temporal differences in viability across the experimental groups. In the control group, sperm survival remained relatively high at 2, 4, and 6 min but showed a decline at 8 min and beyond. Moreover, the DMSO group exhibited a similar trend, while the RES group demonstrated consistently higher sperm survival at all-time points. Strikingly, the AhR¯ group, subjected to the AhR antagonist CH223191, displayed a pronounced and rapid reduction in sperm survival, with complete cessation observed at 10 min.

### Sperm viability assessment

**Figure 5:**
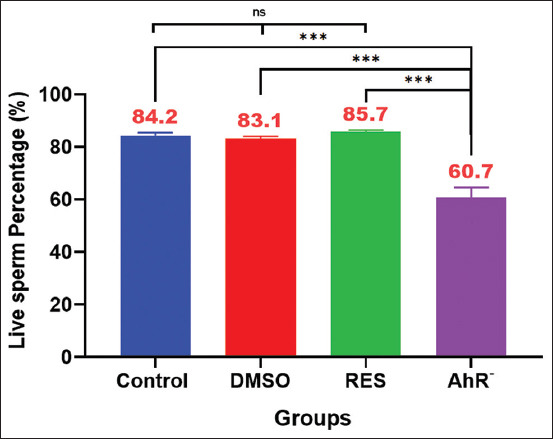
Sperm viability assessment test %. Values are expressed as the means, and error bars represent standard error. DMSO: Administered dimethyl sulfoxide intraperitoneally twice weekly for 60 days. RES: Receiving 100mg/kg resveratrol intraperitoneally twice weekly for 60 days. Ahr¯: Provided with CH223191, intraperitoneally at 10 mg/kg twice weekly for 60 days. Denotes diffe -rences between groups motility, p < 0.05. Ahr=Aryl hydrocarbon receptor, RES=Resveratrol, DMSO=Dimethyl sulfoxide. *p < 0.05, ** p < 0.01, and ***p < 0.001.

In terms of sperm viability (Figure 5), enhanced viability was observed in the RES group compared with the Control group, which served as the baseline. The DMSO group exhibited slightly lower viability than the Control. The AhR¯ group, treated with the AhR antagonist CH223191, exhibited a rapid and pronounced decline in sperm survival, with complete cessation by the 10-min mark.

### Acridine orange staining

**Figure 6:**
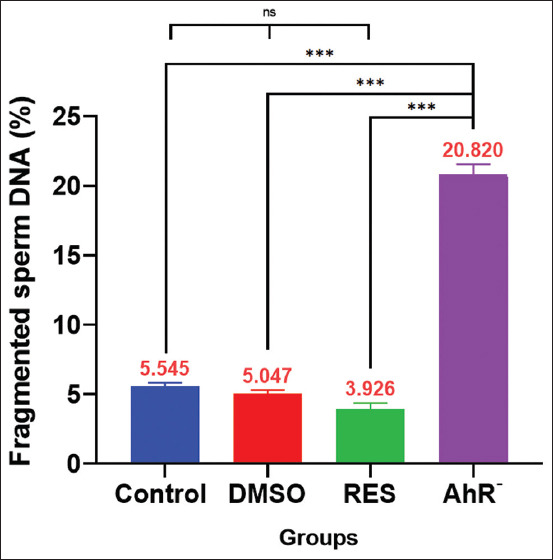
Sperm DNA-fragmentation. Values are expressed as the means, and error bars represent standard error. DMSO: Administered dimethyl sulfoxide intraperitoneally twice weekly for 60 days. RES: Receiving 100mg/kg resveratrol intraperitoneally twice weekly for 60 days. Ahr¯: Provided with CH223191, intraperitoneally at 10 mg/kg twice weekly for 60 days. *Denotes differences between groups motility, p < 0.05. Ahr=Aryl hydrocarbon receptor, RES=Resveratrol, DMSO=Dimethyl sulfoxide. *p < 0.05, ** p < 0.01, and ***p < 0.001.

The results of the sperm DNA fragmentation test using Acridine Orange dye (Figure 6) revealed that the RES group exhibited the lowest mean level of sperm DNA fragmentation, although the reduction was not statistically significant relative to the Control and DMSO groups. Conversely, the AhR¯ group demonstrated significantly increased sperm DNA fragmentation compared with all other groups.

### Redox system evaluation

#### MDA

**Figure 7:**
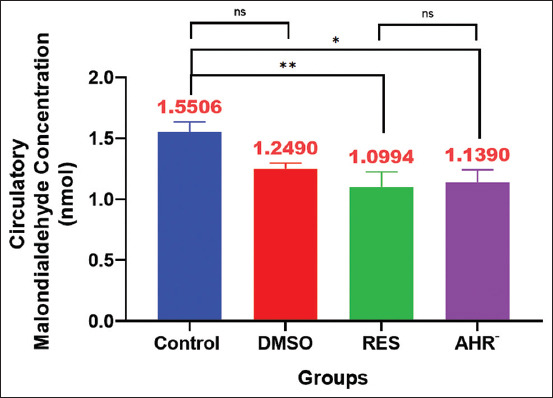
Malondialdehyde. Values are expressed as the means, and error bars represent standard error. DMSO: Administered dimethyl sulfoxide intraperitoneally twice weekly for 60 days. RES: Receiving 100 mg/kg resveratrol intraperitoneally twice weekly for 60 days. Ahr¯: Provided with CH223191, intraperitoneally at 10 mg/kg twice weekly for 60 days. *Denotes differences between groups motility, p < 0.05. Ahr=Aryl hydrocarbon receptor, RES=Resveratrol, DMSO=Dimethyl sulfoxide. *p < 0.05, ** p < 0.01, and ***p < 0.001.

The results of the MDA assay are shown in Figure 7. The RES group demonstrated a significant decrease in MDA levels compared with other groups. The AhR¯ group displayed a significant reduction in TAC compared with the Control (Figure 7).

#### TAC

**Figure 8:**
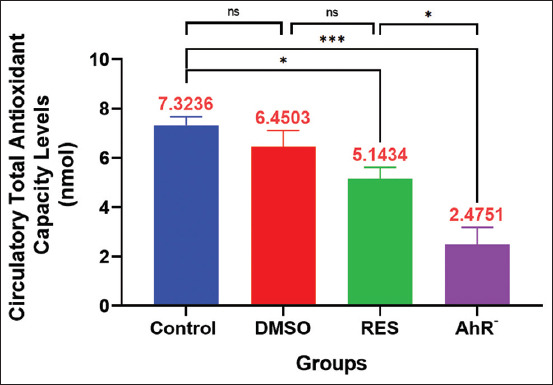
Total antioxidant capacity. Values are expressed as the means, and error bars represent standard error. DMSO: Administered dimethyl sulfoxide intraperitoneally twice weekly for 60 days. RES: Receiving 100mg/kg resveratrol intraperitoneally twice weekly for 60 days. Ahr¯: Provided with CH223191, intraperitoneally at 10 mg/kg twice weekly for 60 days. *Denotes differences between groups motility, p < 0.05. Ahr=Aryl hydrocarbon receptor, RES=Resveratrol, DMSO=Dimethyl sulfoxide. *p < 0.05, ** p < 0.01, and ***p < 0.001.

The TAC results (Figure 8) shed light on the impact of RES and CH223191 on the overall antioxidant defense system. The RES group demonstrated a significant reduction in TAC concentration compared with Control; similarly, the AhR¯ group exhibited a reduced TAC level compared with the Control group and DMSO (Figure 8).

### Gene expression of AhR

**Figure 9:**
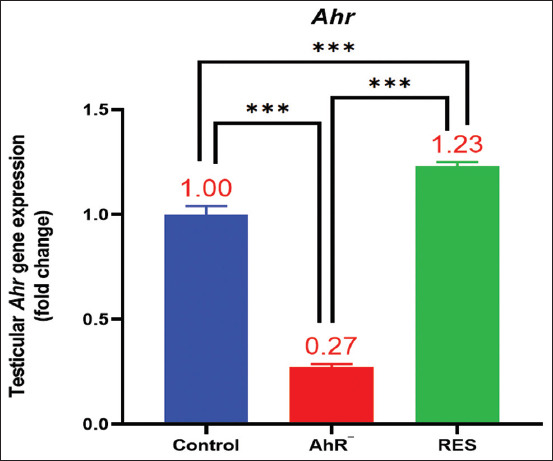
Fold change analysis of Ahr gene expression in response to resveratrol and Ahr antagonist treatments. Values are expressed as the means, and error bars represent standard error. RES: Receiving 100 mg/kg resveratrol intraperitoneally twice weekly for 60 days. Ahr¯: Provided with CH223191, intraperitoneally at 10 mg/kg twice weekly for 60 days. *Denotes differences between groups motility, p < 0.05. Ahr=Aryl hydrocarbon receptor, RES=Resveratrol. *p < 0.05, ** p < 0.01, and ***p < 0.001.

Fold change values reflected the relative alterations in *AhR* gene expression in the RES and AhR¯ groups compared with the Control. The Control group, serving as the baseline with a fold change of 1, establishes a reference point for comparison. In the RES group, gene expression showed an average increase of approximately 23.1%, denoted by a fold change of 1.231, indicating a positive regulatory effect of RES. Conversely, the AhR¯ group exhibited a significant decrease in gene expression, with a fold change of approximately 0.274, indicating a reduction of about 72.6% (Figure 9).

### Histopathological examination of the testes

**Figure 10:**
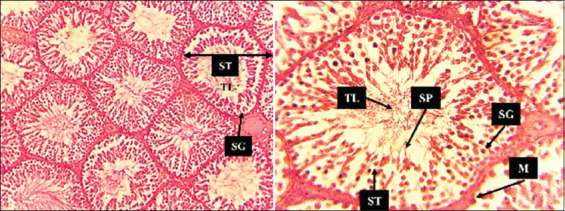
Histological section of control group testicular tissue. Normal histological architecture of testicular tissue. The micrograph shows seminiferous tubules under 10× and 40×, The seminiferous tubule, the tubule lumen, the germ cells of the spermatogenic lineage and prominent among the latter are spermatogonia.

The histopathological study revealed distinct testicular differentiation throughout the course of the study. By scrutinizing the histopathological characteristics of testicular tissue, the results in Figure 10 illustrate a histological section of the Control group, depicting the normal histological architecture of testicular tissue. The observed histological features of the testicular tissue included a thin basement membrane enveloping the seminiferous tubule, which is characteristic of its normal structural composition. The seminiferous tubules exhibited a typical arrangement with sparse interstitial blood vessels. Within the tubular lumens, the presence of mature spermatozoa was noted. The epithelial layer maintained its structural integrity, revealing the presence of Sertoli cells and germ cells representing various stages of spermatogenesis.

**Figure 11:**
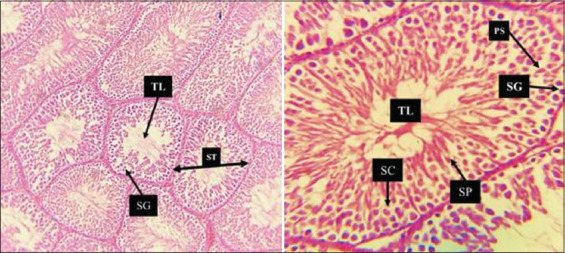
Histological section of resveratrol group testicular tissue. Normal histological architecture of testicular tissue. The micrograph shows seminiferous tubules under 40×. The seminiferous tubule, the tubule lumen, inside the tubule itself is a unique seminiferous epithelium composed of columnar supporting cells called Sertoli cells. Which usually have oval nuclei and distinct nucleoli, and germ cells of the spermatogenic lineage. Prominent among the latter are spermatogonia.

**Figure 12:**
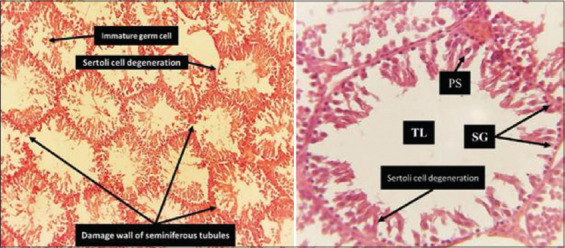
Histological section of Ahr¯ group testicular tissue. Abnormal histological architecture of testicular tissue, the micrograph shows seminiferous tubules under 10× and 40×. The germ cells of the spermatogenic lineage. Prominent among the latter are spermatogonia, diploid cells always located near the basement membrane, and primary sperm cell which are undergoing meiosis closer to the lumen of the tubule lumen.

On the other hand, the histopathological section of the RES group, shown in Figure 11, was normal, as observed in the Control group. Intriguingly, the AhR¯ group, as depicted in Figure 12, exhibited conspicu-ous abnormalities in the histopathological examination that indicated irregularities in tissue structure and function, as particularly evident in the observed deformed seminiferous tubules. The section revealed seminiferous tubule profiles characterized by tubular atrophy (reduced size) and tubular separation, which are notable separations from each other. The epithelial cells of some tubules displayed abnormal attachment, resulting in germinal epithelium disorganization and destruction, accompanied by a reduction in the number of germ cells. In addition, the lumen of these deformed tubules contained immature germ cells and an accumulation of cell fragments within the tubules. Furthermore, interstitial fibrosis and expansion were observed between seminiferous tubules, signifying structural alterations in the testicular tissue of the AhR¯ group.

**Figure 13:**
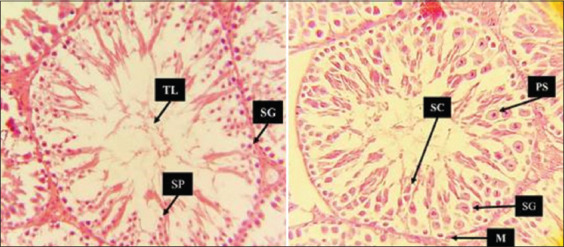
Histological section of dimethyl sulfoxide group testicular tissue. Minor alterations in histological architecture of testicular tissue. The micrograph shows seminiferous tubules under 40×. The germ cells of the spermatogenic lineage, prominent among the latter are spermatogonia, the diploid cells always located near the basement membrane, and primary sperm cell which are undergoing meiosis closer to the lumen of the tubule lumen.

Finally, the results in Figure 13 illustrated that the DMSO group showed normal but minor alterations, including tubular shrinkage in seminiferous tubules, some interstitial expansion between seminiferous tubules, and some basement membrane thickening of the tubules.

## DISCUSSION

The primary aim of this investigation was to unravel the nuanced interactions between RES, CH223191, and AhR, offering insights into their potent -ial implications for the intricate processes govern-ing male reproductive health through a meticulous approach encompassing a range of assessments, including sperm parameters, redox system evaluation, histopathological examination, and gene expression analysis.

When focusing on the histopathological examination of testicular tissue, a crucial aspect of this multifaceted investigation unfolds. Histopathological evaluation revealed distinct alterations among the experimental groups, beginning with the control group, which exhibited normal testicular architecture. The histological section unveils the expected, characteristic architecture of testicular tissue – seminiferous tubules enveloped by a thin basement membrane, exhibiting a typical arrangement with sparse interstitial blood vessels. This extends to the presence of mature spermatozoa within the tubular lumens, maintaining the structural integrity of the epithelial layer with Sertoli cells and germ cells representing various stages of spermatogenesis [36].

The RES group resembled the control group in terms of testicular structure, suggesting that the administration of 100 mg/kg RES twice weekly for 60 days did not cause significant histopathological damage, suggesting a degree of histological preservation of RES on testicular tissue due to its health benefits, due to its anti-inflammatory and antioxidative properties [37]. In general, RES has shown promising results in protecting rat testicular tissue against various forms of damage [27, 38, 39]. RES acts as a direct free radical scavenger, neutralizing reactive species, thereby mitigating oxidative stress and cellular damage associated with aging and disease [40]. In addition, RES has been shown to upregulate the expression of various antioxidant enzymes, including superoxide dismutase, catalase, and glutathione peroxidase [40–42]. These enzymes play a crucial role in the body’s defense against oxidative stress by neutralizing harmful free radicals [43]. RES has also been shown to enhance mitochondrial function and biogenesis since improving mitochondrial function can help reduce ROS production and thus decrease oxidative stress.

On the other hand, a previous study by Meng *et al*. [44] has found that RES inhibits the activation of various signaling pathways that are triggered by oxidative stress, such as the nuclear factor-kappa B pathway, which plays a key role in the regulation of immune and inflammatory responses, and its overactivation can lead to increased oxidative stress. In terms of the Nrf2 pathway, RES has been shown to induce the activation of Nrf2, which is considered a key regulator of cellular resistance to oxidants. On activation, Nrf2 translocates to the nucleus and binds to the antioxidant response element in DNA, leading to the transcription of various antioxidant and detoxifying genes [45].

Previous studies by Casper *et al*. [20] and Xue *et al*. [21] have demonstrated that RES interacts with the AhR, promoting the translocation of AhR to the nucleus and its binding to DNA at responsive elements to regulate gene expression. Interestingly, despite promoting AhR translocation, RES inhibits some subsequent transactivation processes, leading to various forms of cell death, including necrosis, apoptosis, and pyroptosis [22, 23]. This inhibitory effect extends to specific targets such as cytochrome P450 1A1 [24] and interleukin-1β [25, 26]. Moreover, additional research indicates that RES exhibits competitive antagonism for AhR. This implies that RES competes with other ligands for binding to AhR, consequently mitigating their associated toxic and carcinogenic effects. These intricate interactions suggest a potential role for RES in modulating the effects of AhR activation, offering a prospect for safeguarding against the harmful consequences induced by AhR ligands [26].

Overall, AhR contributes to the intricate process of sperm cell development, encompassing distinct phases including the Golgi, Cap, Tail, and Maturation phases, as well as being a crucial factor in spermiation since it plays a role in the sequential development of sperm cells and their eventual release during spermiation [14, 46, 47]. In addition, the AhR is involved in the regulation of Sertoli cells, which are essential for spermatogenesis since Sertoli cells provide nourishment and structural support to developing sperm cells; thus, AhR affects the function of Sertoli cells, thereby influencing spermatogenesis [48, 49]. Furthermore, AhR signaling plays a crucial role in regulating germ cell survival and preventing excessive apoptosis, which is essential for maintaining the requisite pool of germ cells necessary for spermatogenesis.

Previous studies on AhR knockout (AhR^−/−^) mice have demonstrated that the absence of AhR correlates with a reduction in the expression of genes involved in the defense against ROS. The AhR signaling pathway has been implicated as a stress rheostat, influencing spermatogenesis and the histopathological features of the seminiferous epithelium, since the evaluation of AhR−/− males revealed Sertoli cell degeneration, defects in mature sperm, and architectural distortions at stages IV-XII of the seminiferous epithelium [14, 47]. In parallel, our findings align with previous research indicating that RES’s interaction with AhR supports the notion that RES exhibits agonism for AhR and modulates the effects of AhR activation, offering a prospect for improvement in AhR function [26].

In addition, the result of the AhR¯ group (Figure 12), in which CH223191 was administered intraperitoneally at 10 mg/kg twice weekly for 60 days, sheds light on the potential consequences of AhR inhibition, revealing a substantial impact on testicular tissue integrity. The distinct abnormalities observed in the AhR¯ group – seminiferous tubule deformities, reduced size, increased separation between tubules, immature germ cells, and cellular debris – underscore the importance of AhR in maintaining normal testicular tissue architecture.

In a parallel vein, the results shed light on the intricate dynamics involving AhR and RES in the context of sperm health. The examination of sperm DNA, including the sperm DNA fragmentation test, sperm nuclear chromatin condensation, and sperm chromatin maturity, reveals distinctive patterns associated with AhR antagonism and RES treatment. The sperm DNA fragmentation test using Acridine Orange stain evaluates sperm chromatin integrity and maturity due to its selective binding to genetic material, which provides essential insights into the genetic structure of sperm [33, 50]. Acridine Orange distinguishes between normal, well-condensed, and mature sperm nuclei, indicated by green fluorescence when intercalated into native double-stranded DNA. Conversely, red fluorescence denotes chromatin denaturation, suggesting that immature sperm have elevated levels of single-stranded DNA [34].

The results shown in Figure 6 shed light on the intricate impact of AhR modulation and RES intervention on sperm chromatin integrity. Remarkably, despite the RES group demonstrating a non-significant difference compared with the control group, it exhibited a notable reduction in sperm DNA fragmentation. Comparing the findings of the RES group with those of previous studies in the field, the observed reduction in sperm DNA fragmentation aligns with the antioxidative properties attributed to RES, as studied by Gu *et al*. [40], Farkhondeh *et al*. [45], and Revel *et al*. [51], who illustrated that RES scavenges free radicals, enhances antioxidant enzyme expression, and protects against oxidative stress.

Furthermore, RES interacts with AhR, promoting its translocation to the nucleus and its binding to DNA at responsive elements to regulate gene expression [20, 37]. The activation of AhR plays a supportive role in the development of sperm cells, since AhR leads to increased cell proliferation, metabolism, and biosynthesis of lipids, proteins, and nucleotides through the PI3K/Akt pathway [52, 53]. Conversely, studies on AhR^−/−^ mice have shown a decrease in DNA integrity and mitochondrial adenosine triphosphate (ATP) production, as well as alterations in the expression of several genes involved in fatty acid and nucleotide synthesis [54]. Furthermore, AhR plays a critical role in chromatin remodeling and functions through SIRT1 activity, miR expression, and Brahma-related gene 1 regulation [55, 56].

In contrast, the AhR¯ group demonstrated a significant increase in sperm DNA fragmentation compared with all other groups, indicating that strong AhR antagonism by CH223191 may compromise sperm DNA integrity [1, 52, 57].

Our investigation into gene expression, particularly focusing on key genes associated with male reproductive health, in the context of AhR modulation, sheds light on the intricate regulatory mechanisms influencing male reproductive health (Figure 9). The RES group fold change showed an increase, indicating a positive regulatory effect of RES on *AhR* gene expression. In contrast, the AhR¯ group exhibited a significant decrease in gene expression. These findings are consistent with previous research suggesting that RES modulates AhR activity, potentially acting as a partial agonist that influences gene expression dynamics [20–23, 25]. The activation of AhR by RES drives its translocation into the nucleus [58], where it controls the expression of a large number of target genes, including the AhR repressor, detoxifying monooxygenases (CYP1A1 and CYP1B1), and cytokines [24, 59].

Interestingly, studies suggested that RES’s effects on AhR activity can vary depending on its concentration; at low concentrations, RES enhances AhR activity, but at higher concentrations (~200 nM), it acts as an inhibitor. This dual role suggests that RES could have complex effects on cellular pathways involving the AhR [60, 61].

CH223191 is a potent and specific antagonist of the AhR and selectively binds to the ligand-binding domain of AhR, competing with AhR ligands, and causes blockage of AhR signaling pathways, which aligns with the study results and highlights the inhibitory nature of CH223191 on AhR-mediated responses [24].

Commencing discussion with a focus on the physical assessment of epididymal sperms, particularly the parameter of motility (Figure 3), which investigates the intricate dynamics that are critical to male reproductive health. Sperm motility, a cornerstone of sperm function, serves as a vital indicator of reproductive competence [17]. Sperm motility is a complex process regulated by a series of molecular mechanisms that begin with the activation of a soluble adenylyl cyclase in response to changes in intracellular and extracellular conditions, pH, and calcium ions (Ca^²+^) and carbonate ion (HCO_3¯_) concentrations [62]. The activated adenylyl cyclase then catalyzes the conversion of ATP to cyclic AMP (cAMP), a secondary messenger [63, 64]. The increase in cAMP levels leads to the activation of protein kinase A, which phosphorylates several proteins, initiating a phosphorylation cascade. The end result of this cascade is the phosphorylation of the motor protein axonemal dynein. The phosphorylated dynein then induces sliding between the microtubules in the axoneme of the sperm tail, causing the tail to move in a whip-like motion. This flagellar movement propels the sperm cell forward [65, 66].

The study outcomes, derived from various analyses such as histopathological examination of testicular tissue, sperm DNA fragmentation, chromatin condensation, and gene expression analysis focusing on AhR expressions, collectively indicate the promoting effect of RES across all stages of spermatogenesis. Notably, RES demonstrated positive effects on sperm parameters by activating AhR, as evidenced by its impact on ATP catalysis. RES increased the AMP: ATP ratio within the cell, leading to the direct activation of AMPK, a mechanism supported by previous studies by Moon [67] and Salih *et al*. [68].

Conversely, the AhR¯ group showed markedly reduced sperm quality parameters, reinforcing the essential regulatory role of AhR in male reproductive function. The results emphasize the contrasting effects of AhR modulation, where RES positively influences sperm parameters, while AhR inhibition, as observed in the AhR¯ group, leads to a notable decline in sperm assessment. This intricate interplay highlights the significance of AhR in regulating key aspects of spermatogenesis and reinforces the potential therapeutic benefits of RES in male reproductive health.

## CONCLUSION

This study provides compelling evidence that modulation of the AhR through RES and its antagonist CH223191 exerts significant influence on male reproductive health in a rat model. Notably, RES administration resulted in improved sperm motility, survival, and viability, reduced sperm DNA fragmentation, preserved testicular architecture, and upregulated *AhR* gene expression, indicating a protective role mediated through its antioxidative and AhR-modulating effects. In contrast, CH223191-induced AhR inhibition led to marked deterioration in sperm parameters, elevated DNA fragmentation, pronounced testicular histopathological alterations, and significant downregulation of AhR expression, thereby underscoring the receptor’s essential role in testicular function and spermatogenesis.

The strengths of this investigation lie in its multifaceted experimental design, encompassing comprehensive assessments of sperm quality, oxidative stress biomarkers (TAC, MDA), histopathological evaluation, and gene expression analysis. The use of both pharmacological activation and inhibition of AhR provides robust insight into the bidirectional regulatory potential of this receptor in male fertility.

However, several limitations must be acknow-ledged. First, the study was restricted to a single species and dose regimen, which may limit the translatability of findings to humans or other animal models. Second, while the expression of AhR was quantified, downstream signaling pathways and epigenetic regulators were not explored. Third, the sample size per group was modest, which may limit the statistical power for detecting subtle molecular changes.

Future research should focus on delineating the downstream signaling cascades of AhR in testicular tissue, including cross-talk with the PI3K/Akt and Nrf2 pathways. In addition, long-term studies assessing fertility outcomes, progeny health, and potential dose-response effects of RES and CH223191 will further clarify their therapeutic potential. Exploration of AhR modulation in clinical populations with idiopathic infertility or oxidative stress-induced testicular dysfunction may open avenues for targeted interventions.

This study highlights the dual role of AhR in male reproductive physiology and identifies RES as a promising candidate for ameliorating oxidative stress-mediated testicular dysfunction through partial AhR activation.

## AUTHORS’ CONTRIBUTIONS

GSB: Data curation, formal analysis, methodology, writing – Original draft, and writing – review and editing of the manuscript. HFKA: Conceptualization, project administration, supervision, data curation, formal analysis, visualization, and writing – review and editing of the manuscript. Both authors have read and approved the final manuscript.
